# Supplementary material for: Identifying research priorities for patient safety in mental health: an international expert Delphi study
Source: BMJ Open. 2018 Mar 3;8(3):e021361. doi: 10.1136/bmjopen-2017-021361 (PMC5855203; doi:10.1136/bmjopen-2017-021361)
Supplement: Supplementary file 1 [file bmjopen-2017-021361supp001.pdf]

Supplementary file 1

Table 1: Research priorities for patient safety in mental health

No.	Research priority	% Consensus (n=79)
**Governance and improvement**		
**1**	To gain consensus on guidelines on how patient observation should occur and keep patients safe	78.6%
**Restraint**		
**6**	To use quality research to explore how people feel about restraint	71.9%
**7**	To identify important factors in allowing reduction in restrictive practices including restraint and seclusion	90.5%
**8**	To identify the main contributory factors in the use of restraint	85.7%
**9**	To establish the prevalence of restraint and reasons for differences among units	71.4%
**11**	To explore patients' trauma after restraint	78.6%
**12**	To understand why people die from restraint	71.4%
**13**	To identify alternatives to restraint	88.1%
**Children and patient safety**		
**15**	To understand how problems arise and escalate related to self-harm and suicide in children	83.3%
**16**	To explore children's safety when parents with mental health problems become unwell	88.1%
**17**	To explore preventing other harm and secondary damage to children	81.0%
**General safety**		
**18**	To conduct an observational study of adverse events on psychiatric units	85.7%
**19**	To identify what errors or adverse events happen as patients' interface between the medical and mental health system	76.2%
**20**	To investigate preventable errors or adverse events in general in mental health patients	78.2%
**21**	To explore the relationship between therapeutic engagement and patient safety	81.0%

**23**	To explore why people disengage with services on discharge from a mental health unit	73.8%
**24**	To identify the important factors on discharge to keep people safe and comparison across services	88.1%
**25**	To investigate the safe alternatives to admitting someone to hospital	81.0%
**26**	To examine dual diagnosis patients falling through the gap and what can be done to prevent this	71.4%
**Suicide prevention**		
**27**	To conduct a well-controlled study interventions to reduce suicide in outpatients	81.0%
**28**	To compare and contrast community approach, hospital approach and mental health approach in their attempt to reduce suicide	71.4%
**29**	To establish the effectiveness of suicide prevention interventions in large patient samples	83.3%
**30**	To examine suicide prevention in marginal groups	81.0%
**31**	To identify risk factors for suicide on discharge from hospital	78.6%
**32**	To detect people who are at risk of suicide but are not in the mental health system	78.1%
**33**	To identify adverse events that happen to mental health patients above and beyond suicide	81.0%
**Safe environments and culture**		
**39**	To examine the influence of the environment on violent behaviour	76.2%
**40**	To identify environmental factors that indicate a safe environment	81.0%
**41**	To establish the best approach to ensuring safe environments in mental health units	85.7%
**42**	To examine the management of a safe and therapeutic inpatient space	81.0%
**43**	To understand the cultural aspects of safety within an organisation or ward and the major influences in improving the safety culture	83.3%
**44**	To explore the climate and cultural tool use in relation to what influences patient safety culture	75.0%
**45**	To establish best practice guideline for design of an inpatient setting	87.5%
**46**	To identify what constitutes a safer acute ward build	73.8%
**Violence management**		
**47**	To identify positive and negative factors that influence violence management	78.6%

**48**	To understand what prevents violence in patients with mental health conditions	78.6%
**Risk assessment**		
**49**	To understand the nature of the concept of risk assessment	75.0%
**50**	To examine the benefits of a good risk assessments system versus someone who had no risk assessment	78.1%
**Patient perspective or patient centred**		
**53**	To explore the patient perspectives on reasons for their self-harm behaviour	73.8%
**54**	To explore patient perspectives on ways staff can help them manage their self-harm behaviour	73.8%
**56**	To explore the patient perspective on medication safety	90.6%
**57**	To explore patient perspectives on ligature points in an inpatient environment	78.1%
**58**	To explore patient perspectives on their own safety	73.8%
**59**	To explore staff and patient perspective on what determines a safer culture in inpatient setting	87.5%
**60**	To explore perspectives on safety culture in patients who self-harm	90.6%
**61**	To explore patient experience of staff assault on patients	78.1%
**62**	To explore patient experience of complaints after coercive intervention	75.0%
**Decision making and safety**		
**63**	To investigate staff decisions to admit patients presenting with self-harm behaviour	81.3%
**Demographic studies and safety**		
**65**	To evaluate same sex wards compared to mixed sex wards in relation to patient safety	78.1%
**66**	To examine the role of racism in the mental health system in relation to coercive interventions	71.4%
**Psychological trauma**		
**67**	To examine mechanism of trauma and psychological harm associated with inpatient admission	78.1%
**68**	To explore patient experience of coercion in those who've experienced trauma	78.1%
**Safety intervention studies**		

**73**	To conduct trial of different interventions to reduce patient safety incidents compared with other interventions	71.4%
**75**	To establish de-escalation should constitute and how to evaluate it	87.5%
**Safety plans and self-management**		
**76**	To understand specific things patients know regarding their own risk	71.4%
**77**	To identify what constitutes good self-driven individualised safety planning	90.6%
**78**	To conduct a large trial on whether safety plans could improve safety	90.6%
**79**	To investigate personalised model of risk	81.3%
**80**	To understand how patients themselves can contribute to their own safety	100.0%
**81**	To examine the self-management of service users of their own recovery	84.4%
**Staff perspective**		
**89**	To explore the attitudes of staff and its impact on coercive practice	75.0%
**90**	To identify future training of staff in coercive practice	71.9%
**Patients in the community and their safety**		
**95**	To explore what constitutes a place of safety in the community for mentally ill patients	75.0%
**98**	To examine mental health safety in the community	81.3%
**101**	To explore waiting to be seen in accident and emergency rooms and the link to violence	78.1%
**102**	To understand what support is available after discharge	71.9%
**Physical health in mentally ill patients**		
**103**	To examine how improving the quality of life and the physical health of people with severe mental illness	71.4%
**104**	To examine mental health staff engagement with inpatients on their physical health	75.0%
**105**	To compare and contrast physical health hospitals and mental health hospital take on patient safety	71.9%
**107**	To explore of physical health adverse events in mental health patients	81.3%
**108**	Factors related to restraint in mental health patients with physical health problems	71.9%
**109**	Relationship between lack of physical activity in acute settings and dangerous behaviour	75.0%
**Safety in general hospitals for mentally ill patients**		

**111**	To examine safety in mental health patients diagnosed with mental health condition in general medical hospitals	71.9%
**112**	To compare and contrast errors and adverse events in general medical hospitals in patients with mental illness and those without mental illness	71.4%
**Death**		
**115**	To identify reasons for early death in patients with mental health problems	78.1%

**Priorities in bold italics did not come to a consensus*
