# Supplementary material for: Identifying research priorities for patient safety in mental health: an international expert Delphi study
Source: BMJ Open. 2018 Mar 3;8(3):e021361. doi: 10.1136/bmjopen-2017-021361 (PMC5855203; doi:10.1136/bmjopen-2017-021361)
Supplement: Supplementary file 2 [file bmjopen-2017-021361supp002.pdf]

Supplementary file 2

Table 2: Research priorities for patient safety in mental health that did not achieve consensus

No.	Research priority	% Consensus (n=38)
**Governance and improvement**		
**2**	*To examine clinical and cost effectiveness of early patient discharges*	***64.3%***
**3**	*To conduct longitudinal studies on patient records to monitor and improve patient safety*	***57.1%***
**Models of safety**		
**4**	*To understand traditional models of safety and how mental health care works within the model*	***69.0%***
**5**	*To explore philosophical perception of patient safety on patient's rights*	***46.9%***
**Restraint**		
**10**	*To employ a naturalistic study to compare and contrast use of restraint intervention in different countries*	***65.6%***
**14**	*To explore the use of restraint in Black-British men*	***53.6%***
**General safety**		
**22**	*To explore whether mental health first aid has saved a life*	***60.7%***
**Dual diagnosis and safety**		
**34**	*To explore dual diagnosis of substance misuse among psychiatric patients*	***59.4%***
**35**	*To examine the impact on the health and behaviour in dual diagnosed patients*	***53.1%***
**36**	*To conduct a scoping review of range of services available for dual diagnosis patients*	***53.1%***
**37**	*To explore the relationship between specific mental health conditions and substance misuse*	***62.5%***
**38**	*To explore safety incidents in dual diagnosed patients*	***65.6%***
**Research methodology only**		
**51**	*To use mixed methodologies as an research methodology to use*	***57.1%***

**52**	*To use qualitative research methods to explore priorities*	***59.4%***
**Patient perspective or patient centred**		
**55**	*To explore subjective patient experience of coercion*	***67.9%***
**Demographic studies and safety**		
**64**	*To explore LGBT communities with mental health problems and safety incidents*	***59.4%***
**Psychological trauma**		
**69**	*To observe subjective distress and satisfaction in the short-term*	***40.6%***
**Safety intervention studies**		
**70**	*To conduct large longitudinal service wide intervention trial*	***60.7%***
**71**	*To conduct a stepped wedge randomised controlled trial of Safewards*	***64.3%***
**72**	*To implement six core strategies in different settings across countries to understanding outcomes*	***68.8%***
**74**	*To conduct big intervention study on how a patient autonomy model of patient care can be safer*	***60.7%***
**Medication safety**		
**82**	*To examine the impact of medication on the patient*	***59.4%***
**83**	*To conduct randomised controlled observational studies of tapering and withdrawing medication*	***53.1%***
**84**	*To understand about the interaction of multiple medications in relation to safety*	***53.1%***
**85**	*To examine prescription medication assessments in elderly patients with mental health issues*	***50.0%***
**86**	*To explore the polypharmacy and medication management of dual diagnosed patients*	***50.0%***
**87**	*To examine the safety of medication by injection*	***46.9%***
**88**	*To examine the omissions in prescriptions*	***34.4%***
**Staff perspective**		
**91**	*To understand why staff react to events on ward in certain ways*	***65.5%***
**92**	*To explore the physician perception of mental health patients and how they should be managed*	***64.3%***
**93**	*To explore staff attitudes on levels of violent behaviour*	***68.8%***
**Patients in the community and their safety**		
**94**	*To identify places of safety for mentally ill patients in the community*	***68.8%***
**96**	*To examine the impact of discharged patients back into the community with access to guns*	***37.5%***

97	*To understand how the system takes care of the mental health patient in the community*	***64.3%***
99	*To explore safety concerns in the community when patients are withdrawn*	***57.1%***
100	*To assess whether crisis cafes the answer to saving lives rather than being detained in hospital*	***65.6%***
**Physical health in mentally ill patients**		
106	*To examine food as an addiction in mentally ill patients*	***40.6%***
110	*Treatment from staff in mental health patients in emergency or medical situations*	***65.6%***
**Miscellaneous**		
113	*To explore patient personal space and acceptability of body examination*	***43.8%***
114	*To conduct a large European study looking at best practice across numerous countries*	***59.4%***
**Service user involvement**		
116	*To use senior service user researcher to lead mental health and safety work*	***56.3%***
**Incident reporting**		
117	*To compare and contrast recorded and actual observed patient safety incidents in inpatient settings*	***68.8%***

**Priorities in bold italics did not come to a consensus*
