# Supplementary material for: Identifying research priorities for patient safety in mental health: an international expert Delphi study
Source: BMJ Open. 2018 Mar 3;8(3):e021361. doi: 10.1136/bmjopen-2017-021361 (PMC5855203; doi:10.1136/bmjopen-2017-021361)
Supplement: Author's manuscript [file bmjopen-2017-021361.draft_revisions.pdf]

BMJ Open is committed to open peer review. As part of this commitment we make the peer review history of every article we publish publicly available.

When an article is published we post the peer reviewers' comments and the authors' responses online. We also post the versions of the paper that were used during peer review. These are the versions that the peer review comments apply to.

The versions of the paper that follow are the versions that were submitted during the peer review process. They are not the versions of record or the final published versions. They should not be cited or distributed as the published version of this manuscript.

BMJ Open is an open access journal and the full, final, typeset and author-corrected version of record of the manuscript is available on our site with no access controls, subscription charges or pay-per-view fees (http://bmjopen.bmj.com).

If you have any questions on BMJ Open's open peer review process please email info.bmjopen@bmj.com

BMJ Open

Identifying research priorities for patient safety in mental health: an international expert Delphi study

Journal:	*BMJ Open*
Manuscript ID	bmjopen-2017-021361
Article Type:	Research
Date Submitted by the Author:	27-Dec-2017

Complete List of Authors:	Dewa, Lindsay; Imperial College London, School of Public Health; Imperial College London, NIHR Patient Safety Translational Research Centre<br>Murray, Kevin; West London Mental Health NHS Trust<br>Thibaut, Bethan; Imperial College London, School of Public Health<br>Ramtale, Sonny; Imperial College London, NIHR Patient Safety Translational Research Centre<br>Adam, Sheila; Imperial College London, NIHR Patient Safety Translational Research Centre<br>Darzi, Ara; Imperial College London, Department of Surgery and Cancer<br>Archer, Stephanie; NIHR Imperial Patient Safety Translational Research Centre,

Keywords:	patient safety, delphi study, research priorities, MENTAL HEALTH, service users

SCHOLARONE™ Manuscripts

For peer review only

Identifying research priorities for patient safety in mental health: an international expert Delphi study

Lindsay H. Dewa[1,2], Kevin Murray[3], Bethan Thibaut[1], Sonny Christian Ramtale[1], Sheila Adam[1], Ara Darzi[1] and Stephanie Archer[1]

[1]NIHR Imperial Patient Safety Translational Research Centre, Imperial College London, London, UK

[2] School of Public Health, Imperial College London, London, UK

[3]West London Mental Health Trust, London, UK

Correspondence address: Dr Lindsay Dewa, NIHR Imperial Patient Safety Translational Research Centre, Imperial College London, Dr Foster Unit, 3 Dorset Rise, London, EC4Y 8EN, UK, Telephone: +44 (0)20 7594 2114; Fax: (0)207 594 3137; Email: l.dewa@imperial.ac.uk

For peer review only - http://bmjopen.bmj.com/site/about/guidelines.xhtml

ABSTRACT

Objective: Physical healthcare has dominated the patient safety field; research in mental healthcare is not as extensive but findings from physical healthcare cannot be applied to mental healthcare because it delivers specialised care that faces unique challenges. Therefore, a clearer focus and recognition of patient safety in mental health as a distinct research area is still needed. The study aim is to identify future research priorities in the field of patient safety in mental health.

Design: Semi-structured interviews were conducted with the experts to ascertain their views on research priorities in patient safety in mental health. A three-round online Delphi study was used to ascertain consensus on 117 research priority statements.

Setting and participants: Academic and service user experts from the USA, UK, Switzerland, Netherlands, Ireland, Denmark, Finland, Germany, Sweden, Australia, New Zealand and Singapore were included.

Main outcome measures: Agreement in research priorities on a five-point scale.

Results Seventy-nine statements achieved consensus (>70%). Three out of the top six research priorities were patient driven; experts agreed that understanding the patient perspective on safety planning, on self-harm and on medication was important.

Conclusions This is the first international Delphi study to identify research priorities in safety in the mental field as determined by expert academic and service user perspectives. A reasonable consensus was obtained from international perspectives on future research priorities in patient safety in mental health. The research agenda for patient safety in mental health identified here should be informed by patient safety science more broadly and used to further establish this area as a priority in its own right. The safety of mental health patients must have parity with that of physical health patients in order to achieve this.

Strengths and limitations

- Service user experts were involved in both the semi-structured interviews and survey stages.
- The survey response rate was good overall and increased over each Delphi round.
- The selection of experts were mainly from USA and UK, particularly the service users, therefore priorities may be generalizable to only a limited extent.
- The field of patient safety in mental health is relatively new, and as such experts may be more likely to identify themselves as experts in mental health rather than patient safety: experts may therefore have been missed.

Funding

For peer review only - http://bmjopen.bmj.com/site/about/guidelines.xhtml

This work was supported by the National Institute for Health Research (NIHR) Patient Safety Translational Research Centre (PSTRC) via a NIHR programme grant.

Competing interests

None declared

For peer review only

For peer review only - http://bmjopen.bmj.com/site/about/guidelines.xhtml

INTRODUCTION

Patient safety within mental healthcare has not been researched to the same extent as patient safety within physical healthcare [1]. The applicability of findings from physical health cannot simply be applied to mental health because it is a specialised area of care that faces particular challenges [1,2]. For example, some patients receiving mental healthcare may lack mental capacity and may have their freedom limited to a greater or lesser extent. In addition, there is longstanding evidence that mental health services remain under-resourced at a time when need appears to be increasing [3]. Mental health is a relatively neglected area, especially in acknowledging the patient voice in relation to care and safety [4]. Furthermore, stigma and discrimination surrounding mental health issues has the potential to contribute to patient safety being neglected [1].

'Patient safety' in mental health is difficult to define because of the often interrelated understanding of 'disorder' and 'behaviours' for mental health patients within the provision of health care. For example, suicide and self-harm are unsafe behaviours not diagnoses. Therefore patient safety in relation to mental health should mean the avoidance of unintended unsafe or iatrogenic harm associated with mental health care (either an error in inappropriate treatment or an omission to detect unsafe behaviour).

Some types of adverse events that occur due to failure in patient safety in mental healthcare are similar to those in physical healthcare, for example, medication errors, misdiagnosis and accidents. In contrast, unsafe patient behaviours, such as absconding, violence, self-harm and suicide constitute poor outcomes specific to mental healthcare [2]. These may arise from factors inherent in the disorders themselves, giving rise to increased risk to the patient, other patients and to staff, and features of the physical environment and the demanding work environment for staff [1].

Consequently, unsafe patient behaviours within mental healthcare require specialised management strategies distinct to those in physical healthcare. They may require coercive management strategies such as observation, locked wards, seclusion, restraint and enforced medication, which relinquish patients' autonomy. Whilst there has been some attempt to reduce coercion in mental health settings; replaced with de-escalation techniques (e.g. Safewards, active communication) [5], there is still a need to reduce the instances of coercive control further, by staff sharing control with patients over their own care. Indeed, active strategies are in place to help prevent these adverse incidents, with a specific focus on supporting research [6], but a clearer focus and recognition of patient safety in mental health as a distinct research area is still needed.

For peer review only - http://bmjopen.bmj.com/site/about/guidelines.xhtml

Despite the wide range of adverse events, research pertaining to patient safety and mental healthcare is limited. Only three papers have examined priorities in patient safety in a mental health context; they have used a range of research methods including the Delphi technique [1,7,8]. Brickell and colleagues conducted a literature review [1], key informant qualitative interviews and a roundtable event with selected Canadian experts, with the latter published separately [2]. Making safety culture a priority, standardisation of terminology, practices and policies across Canadian mental healthcare were deemed the most important recommendations. Mascherek and Schwappach identified priorities in patient safety in mental healthcare for Switzerland using expert panels, round table discussion and a modified Delphi technique (n=24) [7]. There were nine priority topics but these generally covered iatrogenic safety incidents including diagnostic errors, communication errors and management errors (e.g. aggression management against self and others, unnecessary use of coercive measures). Cowman and colleagues recently conducted a large Delphi study with 2809 staff across 17 countries in Europe to identify current management practices and future priorities relating to violence management in mental health services [8]. Key future priorities in violence management included aetiology, prevention, environmental influence and best management practice.

There are limitations in each of the three studies, and none looked at all aspects of patient safety in mental health internationally. Firstly, two studies focused on priorities in their own countries only, and the results cannot be extrapolated to the wider international platform [1,7]. Secondly in one paper, sample size was low (round 1: n=11; round 2: n=14) and attrition rate high across Delphi rounds [7]. Thirdly, only one study alluded to *research* priorities and this was in the context of violence management only [8]. Finally, and perhaps most importantly, none of the three studies included service users to provide the patient perspective in defining priorities.

The aim of the current study is to use expert consensus building methodology [9] with a group of international academic and service user experts to identify the future research agenda in this area. We refer to “expert service users’ when we are discussing the Delphi participants and the statement generating interviews; we refer to “patients” when we are including the language of the specific statement evaluated.

METHODS

For peer review only - http://bmjopen.bmj.com/site/about/guidelines.xhtml

Design

A Delphi technique was used to build consensus on research priorities for patient safety in mental health (see Figure 1). The Delphi method is an iterative priori process in which a group of expert stakeholders come to a structured consensus view on a particular topic through a number of rounds with controlled feedback [10]. Delphi studies have been successfully conducted to establish research priorities for numerous different topic areas including prison health, mental health and occupational mental health [11–13].

Expert identification and semi-structured interviews

Potential academic experts were identified through a systematic review currently in progress [14] in addition to hand searching of articles and word of mouth. Academic experts satisfied the following inclusion criteria: published at least six articles in patient safety in the mental health field; had at least five years' experience in patient safety in mental health and established reputation in patient safety in mental health, defined as having a high number of citations, a role at a national level or having made a significant impact to the academic field. A provisional list of 15 international academic experts was identified; individuals were contacted via email in the first instance and followed up via phone after a week if there was no response. Participating academic experts were then encouraged to identify other experts through a snowballing technique.

Service user experts were recruited through a UK-based independent third-sector group. Inclusion criteria included: personal experience of mental health services and an ability to comment on patient safety related issues. Service users were excluded if they were unable to provide informed consent due to being too physically or mentally unwell or if they were non-English speaking.

Over a two-month period (February to March 2017), semi-structured telephone interviews were conducted with the academic experts, to ascertain their views on research priorities in patient safety in mental health. Face to face interviews were conducted with service user experts at a place convenient to them. All interviews were recorded. As concrete dimensions of patient safety in mental health do not exist, the topic guide was intentionally broad to bring experts' own knowledge, experience and understanding to the consensus. However, it did cover general questions about known patient safety incidents (e.g. suicide, self-harm etc.) and probing questions on existing patient safety management (e.g. suicide prevention strategies). The topic guide was reviewed by the research team and structured to focus on three main areas: 1) experience of patient safety in mental health; 2) current research in the

For peer review only - http://bmjopen.bmj.com/site/about/guidelines.xhtml

area; 3) research gaps and future research priorities. All audio files from the interviews were transcribed verbatim. Themes were extracted from the transcripts, checked by SA and were used to construct a survey containing statements pertaining to each priority identified.

Delphi round 1

In April 2017, the survey was circulated electronically to a group of international experts. This group comprised the interviewed academic and service user experts, other experts they recommended, and other selected academic experts who had published ≥3 peer reviewed papers [9] in the field of patient safety in mental health. Potential service user experts were approached by email by selected third-sector organisations. The inclusion criteria for service users was the same as the interview criteria. The survey was also advertised on Twitter and in a third-sector newsletter. When completing the survey, experts were asked to indicate anonymously how much they agreed with each statement. Agreement was measured on a 5 point Likert scale: strongly agree, agree, neither agree or disagree, disagree and strongly disagree. The Delphi process concluded when either consensus relating to strongly agree or agree for every individual statement was obtained (>70%) or three rounds were completed [15,16]. As consensus (>70%) was not achieved for all statements in round 1, a further Delphi round was pursued to establish if any additional priorities would achieve consensus.

Delphi round 2

A summary of the collated statement scores that did not reach agreement was then re-circulated to the experts. Experts were given a further two weeks to repeat the survey, choosing whether to amend their scores based on the summary information provided or keep their original score. Once submitted, the new scores were summarised by the researchers and assessed for consensus across the expert group. Consensus was not obtained for all statements, so a third round was pursued to establish any additional research priorities.

Delphi round 3

The distribution and analysis of scores were repeated as in round 2. Experts were given a further two weeks to repeat the survey. We satisfied round 3 criteria and the Delphi rounds ceased.

<Insert Figure 1 here>

For peer review only - http://bmjopen.bmj.com/site/about/guidelines.xhtml

RESULTS

Semi-structured interviews

Nine academic experts participated in the semi-structured interviews (female; n=3). Four experts were based in England; two in the United States and the remaining experts were in Australia, Bahrain and Scotland. The mean number years of experience in the field of patient safety in mental health was 23.22 [SD 8.20] and ranged from 10 to 36 years. Participants had at least 10 publications each in the area of patient safety in mental health (Table 1). Four female service user experts were also interviewed who fulfilled the inclusion criteria (Table 2).

For peer review only

Table 1: Semi-structured interview academic experts[1] (n=9)

Academic	Gender	Academic role	Country	Patient safety in mental health academic expertise					
				Publications	Experience in the field (years)*	Established reputation topic area			
						Preventing suicide	Management of self-harm	Reducing coercive practice	General patient safety in mental health
Academic 1	Female	Associate Professor of Psychiatry and Behavioural Sciences	USA	25	36	X			
Academic 2	Male	Professor of Psychiatry	England	100+	25	X	X		
Academic 3	Male	Adjunct Associate Professor of Psychiatry	USA	21	17	X			
Academic 4	Male	Senior Lecturer	England	10	30	X	X		
Academic 5	Male	Deputy Medical Director	Scotland	10+	10				X
Academic 6	Male	Professor of Nursing	Bahrain/Ireland	15+	20				X
Academic 7	Male	Professor of Mental Health	England	10+	30				X
Academic 8	Female	Associate Professor of Mental Health Nursing	Australia	15	16			X	
Academic 9	Female	Professor of Mental Health Nursing	England	30+	25+				X

* At time of publication

[1] No interests declared

For peer review only

For peer review only - http://bmjopen.bmj.com/site/about/guidelines.xhtml

Table 2: Semi-structured interview service user experts (n=4)

Service user	Gender	Country	Years since last access to mental health services	Type of experience
Service user 1	Female	England	>1	Adult mental health services
Service user 2	Female	England	<1	Adult mental health services
Service user 3	Female	England	>1	Adult mental health services
Service user 4	Female	England	>1	Adult mental health services

In general, both academic and service user experts believed the patient perspective was needed to further understand patient safety in mental health field; however, some academic experts felt this might be difficult in practice, especially with patients with more severe mental health symptoms. Both academics and service users agreed that better understanding of physical health adverse events in mental health patients was a research priority. Academic experts generally agreed more research looking at suicide prevention was needed, but service users spent limited time discussing this. Instead, service user experts explored opportunities for research that were based on their own experience of being a psychiatric inpatient. They spoke about investigating possible alternatives to, and factors directly related to the experience of coercive intervention in addition to alternatives in being admitted to hospital. One hundred and seventeen priority statements were subsequently extracted from the semi-structured interviews (see Supplement 1).

Delphi survey

Forty-two participants took part in the Delphi survey. Two-thirds were academic experts (66.6%) of which 42.9% were female (n=12). Half of the academic experts were based in either the United Kingdom or the United States (50.0%). The remaining experts were in Switzerland, Netherlands, Ireland, Denmark, Finland, Germany, Sweden, Australia, New Zealand and Singapore. The mean years of academic experience in the patient safety in mental health field was 4.8 [SD .57]. Main areas of expertise covered aggression, violence and coercion; violence and suicide prevention; the use of and reduction of restrictive practice including restraint and seclusion, and risk management.

For peer review only - http://bmjopen.bmj.com/site/about/guidelines.xhtml

For peer review only

One third of participants were service user experts (33.3%; n=14) from the United Kingdom. Two-thirds were female (64.3%; n=9) and most had experience with adult mental health services (92.9%; n=13).

In round one, 38/117 statements reached consensus (>70%). The 79 statements that did not achieve consensus were presented to participants again in round two. Three-quarters of participants participated in round two (76.2%; n=32). An additional 35 statements reached consensus. Two participants dropped out before round 3. Subsequently, thirty participants were contacted to take part in round 3. In this final round, twenty-eight participants completed the survey (87.5%). A further six statements reached consensus (>70%). In total, 79 statements achieved consensus.

Main areas of agreement

Patient perspective and safety planning

Understanding how patients can contribute to their own safety emerged as a high research priority across both groups of experts. Patient perspectives on medication safety, safety culture in those who self-harmed and the ways staff could help manage their self-harm were also considered a high priority (90.6%, 90.6% and 73.8% respectively). Experts had agreement on patient-centred research priorities including what constitutes good self-driven safety planning (90.6%), understanding patient's perception of their own risk factors (71.4%) and an exploration into a personalised model of risk (81.3%). Consensus was also obtained on research exploring the patient perspective on staff violence on patients and research on ligature points (both 78.1%).

Physical health in mentally ill patients, mental health and physical health comparison and patient safety in the community

Both academic and service users experts indicated that exploring physical health adverse events in mental health patients should be a priority (81.3%). To a lesser extent, staff engagement with mental health patients about their physical health (75.0%) and comparison of physical and mental health hospitals on patient safety and errors (71.9% and 71.4% respectively) were seen as research priorities. Consensus was also achieved in a research

For peer review only - http://bmjopen.bmj.com/site/about/guidelines.xhtml

priority exploring the relationship between patients waiting to be seen in accident and emergency rooms and the link to general violence (78.1%).

Suicide prevention

The majority of experts, both academic and service users agreed that studies looking at suicide prevention interventions in large samples, marginal groups and outpatients were research priorities in the patient safety in mental health field (83.3%, 81.0% and 81.0% respectively). There was also a preference for research exploring suicide prevention in community samples. For example, the detection of people who are at risk of suicide but not in the mental health system; risk factors for suicide on discharge from hospital and a comparison between the community management, general hospital and psychiatric hospitals on their approach for suicide prevention were deemed future important research areas (78.6%, 78.1% and 71.4% respectively). A need to look at adverse events other than suicide in mentally ill patients was also evident (81.0%)

Safety culture and physical environment

There was clear preference for research on making psychiatric inpatient settings safer for patients. Priorities included the best approach to ensuring a safe environment in mental health units (85.7%), understanding and improving safety culture (83.3%) and identification of environmental factors that indicate a safe environment (81.0%). Likewise, both academic and service user experts wanted to know what environmental factors influence violent incidents and to observe adverse events on the wards (76.2% and 85.7% respectively). Explicit research priorities within this safety culture and environment section included an exploration of the relationship between therapeutic engagement and patient safety, general preventable errors and the safe alternatives to admitting someone to hospital, all of which gained a high-level consensus (81.0%, 78.2% and 81.0% respectively). The establishment of best practice guidelines for the design of inpatient settings was also strongly supported (87.5%).

Restraints

Experts agreed that research into the contributory factors to restraint, specifically in patients with physical health problems was a priority (85.7%). The prevalence and reasons for differences in restraint across units were also a priority (85.7%, 75.0% and 71.4%

For peer review only - http://bmjopen.bmj.com/site/about/guidelines.xhtml

respectively). Two priorities focused on understanding the individual perspective in relation to restraint, including exploration of patient trauma after restraint (78.6%) and how people feel about restraint in general (71.9%). Finally, experts agreed that research was needed to examine factors allowing for reduction in restrictive practice (90.5%) and alternatives to restraint (88.1%).

General safety, risk and violence management

Two research priorities that aimed to examine the nature and benefits of risk assessment (75.0% and 78.1% respectively) were supported by experts. Additionally, experts indicated positive and negative factors that influence violence management and more protective factors for violence in mental health patients were deemed necessary research areas (both 78.6%). Furthermore, experts agreed that the role of racism in relation to coercive interventions should be examined (71.4%). Other proposed priorities with strong agreement included staff decisions to admit patients presenting with self-harm behaviour (87.5%), what de-escalation should look like and how to evaluate it (87.5%) and exploration of staff attitudes on coercive practice (75.0%).

Psychological trauma

The expert group agreed that the mechanism of trauma and psychological harm associated with inpatient admission was a research priority (78.1%). There was also consensus that the patient experience of coercion in those who had experienced trauma should be examined (78.1%).

Children's safety

Experts indicated that research exploring the impact of adverse events for parents on the psychological wellbeing of their children was an important future research area. An understanding of predictive factors in self-harm and suicide in children and an exploration of a child's safety when parents with mental health problems become unwell both achieved consensus (83.3% and 88.1% respectively).

DISCUSSION

For peer review only - http://bmjopen.bmj.com/site/about/guidelines.xhtml

Main results and comparison to other studies

This is the first international Delphi study exploring research priorities in patient safety in mental health from both the academic and service user perspective. Sixty-eight per cent of statements were identified as future research priorities in patient safety in mental health (n=79). The top six research priority areas are shown in box 1; half of these areas were identified by patients for consideration. This suggests a need to shift the thinking about patient safety in mental health services to that of acknowledging the patient voice in relation to their own care and safety. This is in line with other clinical domains that have had success with patient involvement in achieving safer services. For example, some interventions that centre on error prevention through promotion of complex behavioural change and patient involvement have been deemed effective

Thirty-two percent of statements were not seen as priorities. Two research priority statements that concerned research identifying specific places of safety in the community had borderline consensus >65%; but in general, patient safety in community mental health settings were not seen to be a research priority. This is surprising given that most mental health care takes place in the community. Also surprising, considering that this was an international study with respondents from the USA, was the finding that participants disagreed/ strongly disagreed that access to guns in the community after discharge after discharge and omissions in prescriptions were research priorities (37.5% and 34.4%). This may be because the majority of survey experts were from countries where gun violence may not be prevalent in the general community, including among mental health patients.

Research on medication safety is seen as a priority in physical healthcare safety [17]. As such, we expected medication safety to also be identified as a research priority in mental health care; however, this was not the case. Although ‘patient perspectives on medication safety’ had strong consensus (Box 1), the remaining statements related to medication safety were not considered research priorities. Medication safety was not seen as a research priority overall; however, this may have been because the statements focused on minority groups including the elderly and patients with a dual-diagnosis and therefore the statements may not have been applicable to most experts, especially service users.

Box 1: Top six future research priorities that achieved the highest consensus

- Patient contributions to their own safety
- The patient perspective on medication safety

For peer review only - http://bmjopen.bmj.com/site/about/guidelines.xhtml

- Perspectives on safety culture in patients who self-harm
- Good self-driven individualised safety planning
- Safety plans and safety improvement
- Factors in allowing reduction in restrictive practice including restraint and seclusion

In general, statements that proposed longitudinal trials and research focusing on Safewards did not achieve consensus. It is possible that as longitudinal studies are deemed expensive and have added temporal demands [18], academic experts felt they would require a large programme grant and therefore were unattainable as a timely research priority. It is also possible experts felt de-escalation techniques may have had some focus in recent years [5,19] and therefore other areas required more focus going forward. Specifically, the identification of positive, protective and negative factors for violence were still deemed necessary research areas.

Some areas were not endorsed as priorities by the semi-structured interviews, including failure to establish diagnosis, the correct diagnosis and to deliver appropriate treatment. In addition, delays in accessing appropriate care because a bed is not available or service is unavailable, were also not mentioned. This is surprising because it has been considered an important barrier to improving mental healthcare service provision [20]; however, it is possible that experts considered this a patient care or quality improvement issue and not patient safety.

Strengths and limitations

This is the first Delphi study to collate research priorities across *all* aspects of patient safety in mental health internationally, over four continents. A survey sample which was notably larger than another Delphi study in the patient safety-mental health field [7] was obtained. A key strength is the involvement of service user experts in both the semi-structured interviews and survey stages. Another key strength is that all but one interviewed academic experts took part in the survey. The overall survey response rate was good and increased rather

For peer review only - http://bmjopen.bmj.com/site/about/guidelines.xhtml

than decreased over each round (round 2, 76.2%; round 3, 87.5%); therefore, attribution bias was reduced.

However, there are some methodological limitations. Firstly, experts were mainly from Europe, the United States and other high income countries; therefore, the established set of research priorities may only reflect a research agenda for developed countries. Secondly, only English speaking women service users participated in the interviews and therefore priorities for may not be generalizable to the wider service user population. Thirdly, it was not easy to define an academic expert in the patient safety *and* mental health field; unlike another expert consensus building study [9]. This was because the patient safety in mental health field is still relatively new with experts likely to identify themselves as experts in mental health rather than patient safety. It is therefore possible academic experts were missed, unlike in another Delphi study [8]. Finally, participant fatigue was a possible contributory factor towards latter statement completion, particularly due to the large amount of statements (n=117).

Implications

This Delphi study should inform the research agenda for patient safety in mental health field, specifically in the UK, US and other developed countries. It is recommended that academics wishing to pursue research in these areas conduct a comprehensive literature review pertaining to each priority area as a first step. Subsequent studies representing each priority area are then needed to target specific research areas, study types and methodologies. The research findings from these studies should be included in future policy and practice.

This study has included the service user 'voice' when identifying research priorities in patient safety in mental health field. Following this, it would be useful to co-create future research with service users, their families and carers, psychiatric nurses and other mental healthcare professionals with the aim of informing studies with an expert perspective. This is in line with current guidance and the increased expectation to involve a range of experts, including service user experts in the design, production and dissemination of research [21].

CONCLUSION

This is the first expert consensus study to identify research priorities in patient safety in the mental health field. Experts identified what they considered to be the most important areas

For peer review only - http://bmjopen.bmj.com/site/about/guidelines.xhtml

for future research; these include the patient perspective on their mental healthcare, including medication safety, safety planning and self-harm management. Whilst this Delphi study shows consensus in several areas between academic and service user experts, not all of these priorities are firmly on the research agenda for patient safety in mental health.

To establish patient safety in mental health as a research priority in its own right, prospective academic research programmes with appropriate funding are required. This will encourage research that helps detect deterioration of mental health, develops safety interventions and generally improves safety. Whilst mental healthcare provision is increasingly seen as a priority, there is still work to be done; the safety of mental health patients must have parity with patient safety within the physical health field.

Authorship/contribution statement

LD drafted and finalised the protocol, designed the study, led the data collection and analysis and interpreted the data. LD drafted the manuscript and is also the corresponding author.
KM advised on clinical definitions, mental health content interpretation and intellectual content. KM was involved in drafting the manuscript and approved the final version to be published.
BT contributed to data collection, was involved in drafting the manuscript and approved the final version to be published.
SR was involved in drafting the manuscript and approved the final version to be published.
SA advised on clinical definitions, critically reviewed intellectual content and was involved in the drafting and critical appraisal of the manuscript. SA also approved the final manuscript.
AD approved the final manuscript to be published.
SAr contributed to designing the protocol, advised on service user recruitment and critically reviewed intellectual content. SAr was also involved in drafting and critical appraisal of the manuscript.

Ethics approval

The study was approved by the Imperial College Research Ethics Committee.

Data sharing statement

No additional data are available.

Figures

Figure 1: Delphi diagram of study process

For peer review only - http://bmjopen.bmj.com/site/about/guidelines.xhtml

REFERENCES

1 Brickell TA, Nicholls TL, Procyshyn RM, *et al. Patient safety in mental health*. Edmonton: : Canadian Patient Safety Institute and Ontario Hospital Association 2009. http://www.patientsafetyinstitute.ca/english/research/commissionedresearch/mentalhealthandpatientsafety/documents/mental health paper.pdf

2 Brickell T, McLean C. Emerging issues and challenges for improving patient safety in mental health: a qualitative analysis of expert perspectives. *J Patient Saf* 2011;**7**:39–44.

3 The Guardian. NHS mental health funding is still lagging behind, says report. 2016.https://www.theguardian.com/society/2016/may/09/nhs-mental-health-funding-is-still-lagging-behind-says-report (accessed 26 Apr 2017).

4 Prince M, Patel V, Saxena S, *et al.* No health without mental health. *Lancet* 2007;**370**:859–77.

5 Bowers L. Safewards: a new model of conflict and containment on psychiatric wards. *J Psychiatr Ment Health Nurs* 2014;**21**:499–508.

6 HM Government, Department of Health. Preventing suicide in England: A cross-government outcomes strategy to save lives. 2012. www.dh.gov.uk/ (accessed 13 Aug 2017).

7 Mascherek AC, Schwappach DLB. Patient safety priorities in mental healthcare in Switzerland: a modified Delphi study. *BMJ Open* 2016;**6**:e011494.

8 Cowman S, Björkdahl A, Clarke E, *et al.* A descriptive survey study of violence management and priorities among psychiatric staff in mental health services, across seventeen european countries. *BMC Health Serv Res* 2017;**17**.

9 Howell A-M, Burns EM, Hull L, *et al.* International recommendations for national patient safety incident reporting systems: an expert Delphi consensus-building process. *BMJ Qual Saf* 2016;bmjqs-2015-004456.

10 Linstone HA, Turoff M. The Delphi Method - Techniques and applications. *delphi method - Tech Appl* 2002;:1–616.

11 Kouyoumdjian FG, Schuler A, Mcisaac KE, *et al.* Using a Delphi process to define priorities for prison health research in Canada. *BMJ Open* 2016;**6**:1–8.

For peer review only - http://bmjopen.bmj.com/site/about/guidelines.xhtml

12 Owens C, Ley A, Aitken P. Do different stakeholder groups share mental health research priorities? A four-arm Delphi study. *Heal Expect* 2008;**11**:418–31.

13 Bissett M, Ot B. Occupational Therapy Research Priorities in Mental Health. *Occup Ther Heal Care* 2001;**14**:1–19.

14 D'Lima D, Archer S, Thibaut BI, *et al.* A systematic review of patient safety in mental health: a protocol based on the inpatient setting. *Syst Rev* 2016;**5**:203.

15 Fink A, Kosecoff J, Chassin M, *et al.* Consensus Methods: Characteristics and Guidelines for Use. http://ajph.aphapublications.org/doi/pdf/10.2105/AJPH.74.9.979 (accessed 21 Jul 2017).

16 Hasson F, Keeney S, McKenna H. Research guidelines for the Delphi survey technique. *J Adv Nurs* 2000;**32**:1008–15.

17 World Health Organisation (WHO). Global Priorities for Research in Patient Safety (first edition). 2008. http://www.who.int/patientsafety/research/priorities/global_priorities_patient_safety_research.pdf (accessed 21 Jul 2017).

18 Caruana EJ, Roman M, Hernández-Sánchez J, *et al.* Longitudinal studies. *J Thorac Dis* 2015;**7**:E537–40.

19 Bowers L, James K, Quirk A, *et al.* Reducing conflict and containment rates on acute psychiatric wards: The Safewards cluster randomised controlled trial. *Int J Nurs Stud* 2015;**52**:1412–22.

20 British Medical Association. Breaking down barriers – the challenge of improving mental health outcomes. London: 2017.

21 Omeni E, Barnes M, MacDonald D, *et al.* Service user involvement: impact and participation: a survey of service user and staff perspectives. *BMC Health Serv Res* 2014;**14**:491.

For peer review only - http://bmjopen.bmj.com/site/about/guidelines.xhtml

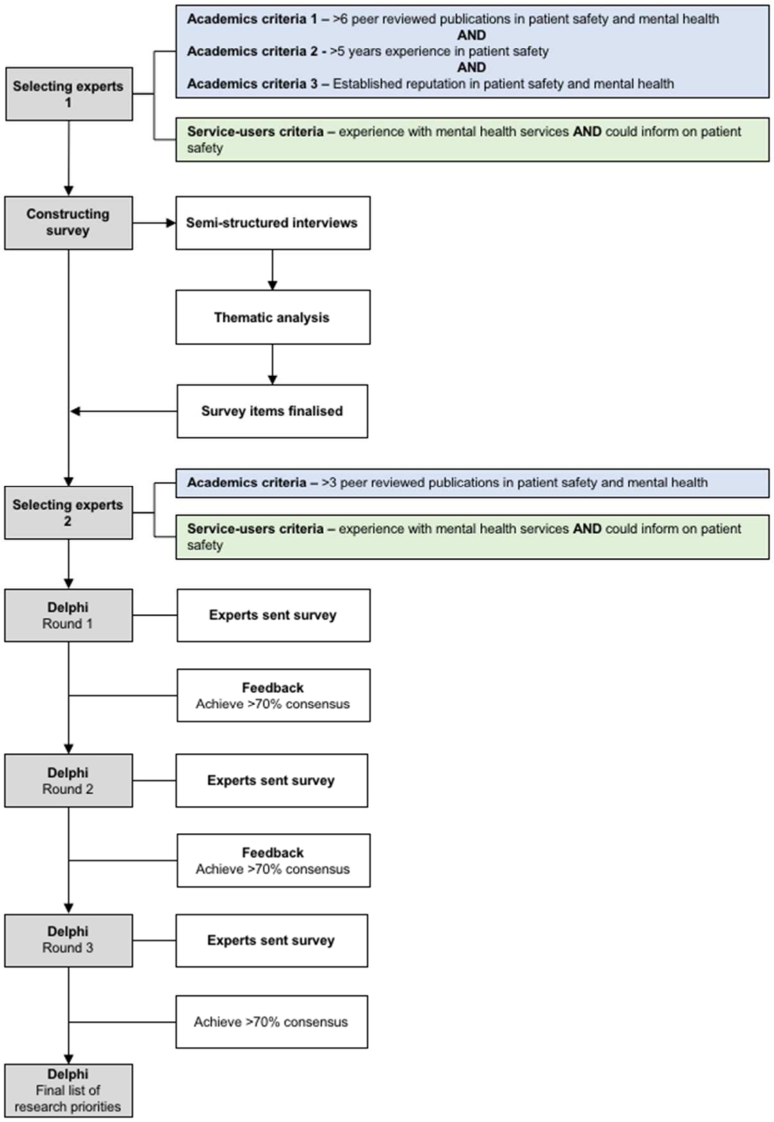

Figure 1: Delphi diagram of study process

190x254mm (96 x 96 DPI)

Supplementary file 1

Table 1: Research priorities for patient safety in mental health

No.	Research priority	% Consensus (n=79)
**Governance and improvement**		
**1**	To gain consensus on guidelines on how patient observation should occur and keep patients safe	78.6%
**2**	*To examine clinical and cost effectiveness of early patient discharges*	***64.3%***
**3**	*To conduct longitudinal studies on patient records to monitor and improve patient safety*	***57.1%***
**Models of safety**		
**4**	*To understand traditional models of safety and how mental health care works within the model*	***69.0%***
**5**	*To explore philosophical perception of patient safety on patient's rights*	***46.9%***
**Restraint**		
**6**	To use quality research to explore how people feel about restraint	71.9%
**7**	To identify important factors in allowing reduction in restrictive practices including restraint and seclusion	90.5%
**8**	To identify the main contributory factors in the use of restraint	85.7%
**9**	To establish the prevalence of restraint and reasons for differences among units	71.4%
**10**	*To employ a naturalistic study to compare and contrast use of restraint intervention in different countries*	***65.6%***
**11**	To explore patients' trauma after restraint	78.6%
**12**	To understand why people die from restraint	71.4%
**13**	To identify alternatives to restraint	88.1%
**14**	*To explore the use of restraint in Black-British men*	***53.6%***
**Children and patient safety**		
**15**	To understand how problems arise and escalate related to self-harm and suicide in children	83.3%
**16**	To explore children's safety when parents with mental health problems become unwell	88.1%

For peer review only

**17**	To explore preventing other harm and secondary damage to children	81.0%
**General safety**		
**18**	To conduct an observational study of adverse events on psychiatric units	85.7%
**19**	To identify what errors or adverse events happen as patients' interface between the medical and mental health system	76.2%
**20**	To investigate preventable errors or adverse events in general in mental health patients	78.2%
**21**	To explore the relationship between therapeutic engagement and patient safety	81.0%
**22**	*To explore whether mental health first aid has saved a life*	***60.7%***
**23**	To explore why people disengage with services on discharge from a mental health unit	73.8%
**24**	To identify the important factors on discharge to keep people safe and comparison across services	88.1%
**25**	To investigate the safe alternatives to admitting someone to hospital	81.0%
**26**	To examine dual diagnosis patients falling through the gap and what can be done to prevent this	71.4%
**Suicide prevention**		
**27**	To conduct a well-controlled study interventions to reduce suicide in outpatients	81.0%
**28**	To compare and contrast community approach, hospital approach and mental health approach in their attempt to reduce suicide	71.4%
**29**	To establish the effectiveness of suicide prevention interventions in large patient samples	83.3%
**30**	To examine suicide prevention in marginal groups	81.0%
**31**	To identify risk factors for suicide on discharge from hospital	78.6%
**32**	To detect people who are at risk of suicide but are not in the mental health system	78.1%
**33**	To identify adverse events that happen to mental health patients above and beyond suicide	81.0%
**Dual diagnosis and safety**		
**34**	*To explore dual diagnosis of substance misuse among psychiatric patients*	***59.4%***
**35**	*To examine the impact on the health and behaviour in dual diagnosed patients*	***53.1%***

For peer review only - http://bmjopen.bmj.com/site/about/guidelines.xhtml

**36**	*To conduct a scoping review of range of services available for dual diagnosis patients*	***53.1%***
**37**	*To explore the relationship between specific mental health conditions and substance misuse*	***62.5%***
**38**	*To explore safety incidents in dual diagnosed patients*	***65.6%***
**Safe environments and culture**		
**39**	To examine the influence of the environment on violent behaviour	76.2%
**40**	To identify environmental factors that indicate a safe environment	81.0%
**41**	To establish the best approach to ensuring safe environments in mental health units	85.7%
**42**	To examine the management of a safe and therapeutic inpatient space	81.0%
**43**	To understand the cultural aspects of safety within an organisation or ward and the major influences in improving the safety culture	83.3%
**44**	To explore the climate and cultural tool use in relation to what influences patient safety culture	75.0%
**45**	To establish best practice guideline for design of an inpatient setting	87.5%
**46**	To identify what constitutes a safer acute ward build	73.8%
**Violence management**		
**47**	To identify positive and negative factors that influence violence management	78.6%
**48**	To understand what prevents violence in patients with mental health conditions	78.6%
**Risk assessment**		
**49**	To understand the nature of the concept of risk assessment	75.0%
**50**	To examine the benefits of a good risk assessments system versus someone who had no risk assessment	78.1%
**Research methodology only**		
**51**	*To use mixed methodologies as an research methodology to use*	***57.1%***
**52**	*To use qualitative research methods to explore priorities*	***59.4%***
**Patient perspective or patient centred**		
**53**	To explore the patient perspectives on reasons for their self-harm behaviour	73.8%
**54**	To explore patient perspectives on ways staff can help them manage their self-harm behaviour	73.8%
**55**	*To explore subjective patient experience of coercion*	***67.9%***

For peer review only

**56**	To explore the patient perspective on medication safety	90.6%
**57**	To explore patient perspectives on ligature points in an inpatient environment	78.1%
**58**	To explore patient perspectives on their own safety	73.8%
**59**	To explore staff and patient perspective on what determines a safer culture in inpatient setting	87.5%
**60**	To explore perspectives on safety culture in patients who self-harm	90.6%
**61**	To explore patient experience of staff assault on patients	78.1%
**62**	To explore patient experience of complaints after coercive intervention	75.0%
**Decision making and safety**		
**63**	To investigate staff decisions to admit patients presenting with self-harm behaviour	81.3%
**Demographic studies and safety**		
**64**	*To explore LGBT communities with mental health problems and safety incidents*	***59.4%***
**65**	To evaluate same sex wards compared to mixed sex wards in relation to patient safety	78.1%
**66**	To examine the role of racism in the mental health system in relation to coercive interventions	71.4%
**Psychological trauma**		
**67**	To examine mechanism of trauma and psychological harm associated with inpatient admission	78.1%
**68**	To explore patient experience of coercion in those who've experienced trauma	78.1%
**69**	*To observe subjective distress and satisfaction in the short-term*	***40.6%***
**Safety intervention studies**		
**70**	*To conduct large longitudinal service wide intervention trial*	***60.7%***
**71**	*To conduct a stepped wedge randomised controlled trial of Safewards*	***64.3%***
**72**	*To implement six core strategies in different settings across countries to understanding outcomes*	***68.8%***
**73**	To conduct trial of different interventions to reduce patient safety incidents compared with other interventions	71.4%
**74**	*To conduct big intervention study on how a patient autonomy model of patient care can be safer*	***60.7%***
**75**	To establish de-escalation should constitute and how to evaluate it	87.5%

For peer review only - http://bmjopen.bmj.com/site/about/guidelines.xhtml

**Safety plans and self-management**		
**76**	To understand specific things patients know regarding their own risk	71.4%
**77**	To identify what constitutes good self-driven individualised safety planning	90.6%
**78**	To conduct a large trial on whether safety plans could improve safety	90.6%
**79**	To investigate personalised model of risk	81.3%
**80**	To understand how patients themselves can contribute to their own safety	100.0%
**81**	To examine the self-management of service users of their own recovery	84.4%
**Medication safety**		
**82**	*To examine the impact of medication on the patient*	***59.4%***
**83**	*To conduct randomised controlled observational studies of tapering and withdrawing medication*	***53.1%***
**84**	*To understand about the interaction of multiple medications in relation to safety*	***53.1%***
**85**	*To examine prescription medication assessments in elderly patients with mental health issues*	***50.0%***
**86**	*To explore the polypharmacy and medication management of dual diagnosed patients*	***50.0%***
**87**	*To examine the safety of medication by injection*	***46.9%***
**88**	*To examine the omissions in prescriptions*	***34.4%***
**Staff perspective**		
**89**	To explore the attitudes of staff and its impact on coercive practice	75.0%
**90**	To identify future training of staff in coercive practice	71.9%
**91**	*To understand why staff react to events on ward in certain ways*	***65.5%***
**92**	*To explore the physician perception of mental health patients and how they should be managed*	***64.3%***
**93**	*To explore staff attitudes on levels of violent behaviour*	***68.8%***
**Patients in the community and their safety**		
**94**	*To identify places of safety for mentally ill patients in the community*	***68.8%***
**95**	To explore what constitutes a place of safety in the community for mentally ill patients	75.0%
**96**	*To examine the impact of discharged patients back into the community with access to guns*	***37.5%***

For peer review only

**97**	*To understand how the system takes care of the mental health patient in the community*	***64.3%***
**98**	To examine mental health safety in the community	81.3%
**99**	*To explore safety concerns in the community when patients are withdrawn*	***57.1%***
**100**	*To assess whether crisis cafes the answer to saving lives rather than being detained in hospital*	***65.6%***
**101**	To explore waiting to be seen in accident and emergency rooms and the link to violence	78.1%
**102**	To understand what support is available after discharge	71.9%
**Physical health in mentally ill patients**		
**103**	To examine how improving the quality of life and the physical health of people with severe mental illness	71.4%
**104**	To examine mental health staff engagement with inpatients on their physical health	75.0%
**105**	To compare and contrast physical health hospitals and mental health hospital take on patient safety	71.9%
**106**	*To examine food as an addiction in mentally ill patients*	***40.6%***
**107**	To explore of physical health adverse events in mental health patients	81.3%
**108**	Factors related to restraint in mental health patients with physical health problems	71.9%
**109**	Relationship between lack of physical activity in acute settings and dangerous behaviour	75.0%
**110**	*Treatment from staff in mental health patients in emergency or medical situations*	***65.6%***
**Safety in general hospitals for mentally ill patients**		
**111**	To examine safety in mental health patients diagnosed with mental health condition in general medical hospitals	71.9%
**112**	To compare and contrast errors and adverse events in general medical hospitals in patients with mental illness and those without mental illness	71.4%
**Miscellaneous**		
**113**	*To explore patient personal space and acceptability of body examination*	***43.8%***
**114**	*To conduct a large European study looking at best practice across numerous countries*	***59.4%***

For peer review only

**Death**		
**115**	To identify reasons for early death in patients with mental health problems	78.1%
**Service user involvement**		
**116**	*To use senior service user researcher to lead mental health and safety work*	***56.3%***
**Incident reporting**		
**117**	*To compare and contrast recorded and actual observed patient safety incidents in inpatient settings*	***68.8%***

*Priorities in bold italics did not come to a consensus

For peer review only

BMJ Open

Identifying research priorities for patient safety in mental health: an international expert Delphi study

Journal:	*BMJ Open*
Manuscript ID	bmjopen-2017-021361.R1
Article Type:	Research
Date Submitted by the Author:	02-Feb-2018
Complete List of Authors:	Dewa, Lindsay; Imperial College London, School of Public Health; Imperial College London, NIHR Patient Safety Translational Research Centre<br>Murray, Kevin; West London Mental Health NHS Trust<br>Thibaut, Bethan; Imperial College London, School of Public Health<br>Ramtale, Sonny; Imperial College London, NIHR Patient Safety Translational Research Centre<br>Adam, Sheila; Imperial College London, NIHR Patient Safety Translational Research Centre<br>Darzi, Ara; Imperial College London, Department of Surgery and Cancer<br>Archer, Stephanie; NIHR Imperial Patient Safety Translational Research Centre,
**Primary Subject Heading**:	Mental health
Secondary Subject Heading:	Mental health
Keywords:	patient safety, delphi study, research priorities, MENTAL HEALTH, service users

SCHOLARONE™
Manuscripts

Identifying research priorities for patient safety in mental health: an international expert Delphi study

Lindsay H. Dewa[1,2], Kevin Murray[3], Bethan Thibaut[1], Sonny Christian Ramtale[1], Sheila Adam[1], Ara Darzi[1] and Stephanie Archer[1]

[1]NIHR Imperial Patient Safety Translational Research Centre, Imperial College London, London, UK

[2] School of Public Health, Imperial College London, London, UK

[3]West London Mental Health Trust, London, UK

Correspondence address: Dr Lindsay Dewa, NIHR Imperial Patient Safety Translational Research Centre, Imperial College London, School of Public Health, Reynolds Building, St Dunstan's Road, London, W6 8RP, UK, Telephone: +44 (0)20 7594 0815, Email: l.dewa@imperial.ac.uk

For peer review only

For peer review only - http://bmjopen.bmj.com/site/about/guidelines.xhtml

ABSTRACT

Objective: Physical healthcare has dominated the patient safety field; research in mental healthcare is not as extensive but findings from physical healthcare cannot be applied to mental healthcare because it delivers specialised care that faces unique challenges. Therefore, a clearer focus and recognition of patient safety in mental health as a distinct research area is still needed. The study aim is to identify future research priorities in the field of patient safety in mental health.

Design: Semi-structured interviews were conducted with the experts to ascertain their views on research priorities in patient safety in mental health. A three-round online Delphi study was used to ascertain consensus on 117 research priority statements.

Setting and participants: Academic and service user experts from the USA, UK, Switzerland, Netherlands, Ireland, Denmark, Finland, Germany, Sweden, Australia, New Zealand and Singapore were included.

Main outcome measures: Agreement in research priorities on a five-point scale.

Results Seventy-nine statements achieved consensus (>70%). Three out of the top six research priorities were patient driven; experts agreed that understanding the patient perspective on safety planning, on self-harm and on medication was important.

Conclusions This is the first international Delphi study to identify research priorities in safety in the mental field as determined by expert academic and service user perspectives. A reasonable consensus was obtained from international perspectives on future research priorities in patient safety in mental health; however, the patient perspective on their mental healthcare is a priority. The research agenda for patient safety in mental health identified here should be informed by patient safety science more broadly and used to further establish this area as a priority in its own right. The safety of mental health patients must have parity with that of physical health patients in order to achieve this.

Strengths and limitations

- Service user experts were involved in both the semi-structured interviews and survey stages.
- The survey response rate was good overall and increased over each Delphi round.
- The selection of experts were mainly from USA and UK, particularly the service users, therefore priorities may be generalizable to only a limited extent.
- The field of patient safety in mental health is relatively new, and as such experts may be more likely to identify themselves as experts in mental health rather than patient safety: experts may therefore have been missed.

For peer review only - http://bmjopen.bmj.com/site/about/guidelines.xhtml

Funding

This work was supported by the National Institute for Health Research (NIHR) Patient Safety Translational Research Centre (PSTRC) via a NIHR programme grant.

Competing interests

None declared

For peer review only

INTRODUCTION

Patient safety within mental healthcare has not been researched to the same extent as patient safety within physical healthcare [1]. The applicability of findings from physical health cannot simply be applied to mental health because it is a specialised area of care that faces particular challenges [1,2]. For example, some patients receiving mental healthcare may lack mental capacity and may have their freedom limited to a greater or lesser extent. In addition, there is longstanding evidence that mental health services remain under-resourced at a time when need appears to be increasing [3]. Mental health is a relatively neglected area, especially in acknowledging the patient voice in relation to care and safety [4]. Furthermore, stigma and discrimination surrounding mental health issues has the potential to contribute to patient safety being neglected [1].

'Patient safety' in mental health is difficult to define because of the often interrelated understanding of 'disorder' and 'behaviours' for mental health patients within the provision of health care. For example, suicide and self-harm are unsafe behaviours not diagnoses. Therefore patient safety in relation to mental health should mean the avoidance of unintended unsafe or iatrogenic harm associated with mental health care (either an error in inappropriate treatment or an omission to detect unsafe behaviour).

Some types of adverse events that occur due to failure in patient safety in mental healthcare are similar to those in physical healthcare, for example, medication errors, misdiagnosis and accidents. In contrast, unsafe patient behaviours, such as absconding, violence, self-harm and suicide constitute poor outcomes specific to mental healthcare [2]. These may arise from factors inherent in the disorders themselves, giving rise to increased risk to the patient, other patients and to staff, and features of the physical environment and the demanding work environment for staff [1].

Consequently, unsafe patient behaviours within mental healthcare require specialised management strategies distinct to those in physical healthcare. They may require coercive management strategies such as observation, locked wards, seclusion, restraint and enforced medication, which relinquish patients' autonomy. Whilst there has been some attempt to reduce coercion in mental health settings; replaced with de-escalation techniques (e.g. Safewards, active communication) [5], there is still a need to reduce the instances of coercive control further, by staff sharing control with patients over their own care. Indeed, active strategies are in place to help prevent these adverse incidents, with a specific focus on supporting research [6], but a clearer focus and recognition of patient safety in mental health as a distinct research area is still needed.

For peer review only - http://bmjopen.bmj.com/site/about/guidelines.xhtml

Despite the wide range of adverse events, research pertaining to patient safety and mental healthcare is limited. Only three papers have examined priorities in patient safety in a mental health context; they have used a range of research methods including the Delphi technique [1,7,8]. Brickell and colleagues conducted a literature review [1], key informant qualitative interviews and a roundtable event with selected Canadian experts, with the latter published separately [2]. Making safety culture a priority, standardisation of terminology, practices and policies across Canadian mental healthcare were deemed the most important recommendations. Mascherek and Schwappach identified priorities in patient safety in mental healthcare for Switzerland using expert panels, round table discussion and a modified Delphi technique (n=24) [7]. There were nine priority topics but these generally covered iatrogenic safety incidents including diagnostic errors, communication errors and management errors (e.g. aggression management against self and others, unnecessary use of coercive measures). Cowman and colleagues recently conducted a large Delphi study with 2809 staff across 17 countries in Europe to identify current management practices and future priorities relating to violence management in mental health services [8]. Key future priorities in violence management included aetiology, prevention, environmental influence and best management practice.

There are limitations in each of the three studies, and none looked at all aspects of patient safety in mental health internationally. Firstly, two studies focused on priorities in their own countries only, and the results cannot be extrapolated to the wider international platform [1,7]. Secondly in one paper, sample size was low (round 1: n=11; round 2: n=14) and attrition rate high across Delphi rounds [7]. Thirdly, only one study alluded to *research* priorities and this was in the context of violence management only [8]. Finally, and perhaps most importantly, none of the three studies included service users to provide the patient perspective in defining priorities.

The aim of the current study is to use expert consensus building methodology [9] with a group of international academic and service user experts to identify the future research agenda in this area. We refer to “expert service users’ when we are discussing the Delphi participants and the statement generating interviews; we refer to “patients” when we are including the language of the specific statement evaluated.

METHODS

For peer review only - http://bmjopen.bmj.com/site/about/guidelines.xhtml

Design

A Delphi technique was used to build consensus on research priorities for patient safety in mental health (see Figure 1). The Delphi method is an iterative priori process in which a group of expert stakeholders come to a structured consensus view on a particular topic through a number of rounds with controlled feedback [10]. Delphi studies have been successfully conducted to establish research priorities for numerous different topic areas including prison health, mental health and occupational mental health [11–13].

Expert identification and semi-structured interviews

Potential academic experts were identified through a systematic review currently in progress [14] in addition to hand searching of articles and word of mouth. Academic experts satisfied the following inclusion criteria: published at least six articles in patient safety in the mental health field; had at least five years' experience in patient safety in mental health and established reputation in patient safety in mental health, defined as having a high number of citations, a role at a national level or having made a significant impact to the academic field. A provisional list of 15 international academic experts was identified; individuals were contacted via email in the first instance and followed up via phone after a week if there was no response. Participating academic experts were then encouraged to identify other experts through a snowballing technique.

Service user experts were recruited through a UK-based independent third-sector group. Inclusion criteria included: personal experience of mental health services and an ability to comment on patient safety related issues. Service users were excluded if they were unable to provide informed consent due to being too physically or mentally unwell or if they were non-English speaking.

Over a two-month period (February to March 2017), semi-structured telephone interviews were conducted with the academic experts, to ascertain their views on research priorities in patient safety in mental health. Face to face interviews were conducted with service user experts at a place convenient to them. All interviews were recorded. As concrete dimensions of patient safety in mental health do not exist, the topic guide was intentionally broad to bring experts' own knowledge, experience and understanding to the consensus. However, it did cover general questions about known patient safety incidents (e.g. suicide, self-harm etc.) and probing questions on existing patient safety management (e.g. suicide prevention strategies). The topic guide was reviewed by the research team and structured to focus on three main areas: 1) experience of patient safety in mental health; 2) current research in the

For peer review only - http://bmjopen.bmj.com/site/about/guidelines.xhtml

For peer review only

area; 3) research gaps and future research priorities. All audio files from the interviews were transcribed verbatim. Themes were extracted from the transcripts, checked by SA and were used to construct a survey containing statements pertaining to each priority identified.

Delphi round 1

In April 2017, the survey was circulated electronically to a group of international experts. This group comprised the interviewed academic and service user experts, other experts they recommended, and other selected academic experts who had published ≥3 peer reviewed papers [9] in the field of patient safety in mental health. Potential service user experts were approached by email by selected third-sector organisations. The inclusion criteria for service users was the same as the interview criteria. The survey was also advertised on Twitter and in a third-sector newsletter. When completing the survey, experts were asked to indicate anonymously how much they agreed with each statement. Agreement was measured on a 5 point Likert scale: strongly agree, agree, neither agree or disagree, disagree and strongly disagree. The Delphi process concluded when either consensus relating to strongly agree or agree for every individual statement was obtained (>70%) or three rounds were completed, in line with guidance [15,16]. As consensus (>70%) was not achieved for all statements in round 1, a further Delphi round was pursued to establish if any additional priorities would achieve consensus.

Delphi round 2

Group feedback and a summary of the collated statement scores that did not reach agreement was then circulated to the experts. Experts were given a further two weeks to repeat the survey, choosing whether to amend their scores based on the summary information provided or keep their original score. Once submitted, the new scores were summarised by the researchers and assessed for consensus across the expert group. Consensus was not obtained for all statements, so a third round was pursued to establish any additional research priorities.

Delphi round 3

The distribution and analysis of scores were repeated as in round 2. Experts were given a further two weeks to repeat the survey. We satisfied round 3 criteria and the Delphi rounds ceased.

<Insert Figure 1 here>

For peer review only - http://bmjopen.bmj.com/site/about/guidelines.xhtml

RESULTS

Semi-structured interviews

Nine academic experts participated in the semi-structured interviews (female; n=3). Four experts were based in England; two in the United States and the remaining experts were in Australia, Bahrain and Scotland. The mean number years of experience in the field of patient safety in mental health was 23.22 [SD 8.20] and ranged from 10 to 36 years. Participants had at least 10 publications each in the area of patient safety in mental health (Table 1). Four female service user experts were also interviewed who fulfilled the inclusion criteria (Table 2).

For peer review only

Table 1: Semi-structured interview academic experts[1] (n=9)

Academic	Gender	Academic role	Country	Patient safety in mental health academic expertise					
				Publications	Experience in the field (years)*	Established reputation topic area			
						Preventing suicide	Management of self-harm	Reducing coercive practice	General patient safety in mental health
Academic 1	Female	Associate Professor of Psychiatry and Behavioural Sciences	USA	25	36	X			
Academic 2	Male	Professor of Psychiatry	England	100+	25	X	X		
Academic 3	Male	Adjunct Associate Professor of Psychiatry	USA	21	17	X			
Academic 4	Male	Senior Lecturer	England	10	30	X	X		
Academic 5	Male	Deputy Medical Director	Scotland	10+	10				X
Academic 6	Male	Professor of Nursing	Bahrain/Irela nd	15+	20				X
Academic 7	Male	Professor of Mental Health	England	10+	30				X
Academic 8	Female	Associate Professor of Mental Health Nursing	Australia	15	16			X	
Academic 9	Female	Professor of Mental Health Nursing	England	30+	25+				X

* At time of publication

[1] No interests declared

For peer review only

For peer review only - http://bmjopen.bmj.com/site/about/guidelines.xhtml

Table 2: Semi-structured interview service user experts (n=4)

Service user	Gender	Country	Years since last access to mental health services	Type of experience
Service user 1	Female	England	>1	Adult mental health services
Service user 2	Female	England	<1	Adult mental health services
Service user 3	Female	England	>1	Adult mental health services
Service user 4	Female	England	>1	Adult mental health services

In general, both academic and service user experts believed the patient perspective was needed to further understand patient safety in mental health field; however, some academic experts felt this might be difficult in practice, especially with patients with more severe mental health symptoms. Experts agreed that better understanding of physical health adverse events in mental health patients was a research priority. Academic experts generally agreed more research looking at suicide prevention was needed, but service users spent limited time discussing this. Instead, service user experts explored opportunities for research that were based on their own experience of being a psychiatric inpatient. They spoke about investigating possible alternatives to, and factors directly related to the experience of coercive intervention in addition to alternatives in being admitted to hospital. One hundred and seventeen priority statements were subsequently extracted from the semi-structured interviews (see Supplement 1 and 2).

For peer review only

Delphi survey

Forty-two participants took part in the Delphi survey. Two-thirds were academic experts (n=28; 66.6%) of which 42.9% were female (n=12). Half of the academic experts were based in either the United Kingdom or the United States (n=14; 50.0%). The remaining experts were in Switzerland, Netherlands, Ireland, Denmark, Finland, Germany, Sweden, Australia, New Zealand and Singapore. The mean years of academic experience in the patient safety in mental health field was 4.8 [SD .57]. Main areas of expertise covered aggression, violence and coercion; violence and suicide prevention; the use of and reduction of restrictive practice including restraint and seclusion, and risk management.

For peer review only - http://bmjopen.bmj.com/site/about/guidelines.xhtml

One third of participants were service user experts (33.3%; n=14) from the United Kingdom. Two-thirds were female (64.3%; n=9) and most had experience with adult mental health services (92.9%; n=13).

In round one, 38/117 statements reached consensus (>70%). The two expert groups (service users and academics) showed modest agreement in their responses (r=.25 p<.01). The 79 statements that did not achieve consensus were presented to participants again in round two. Three-quarters of participants participated in round two (76.2%; n=32). An additional 35 statements reached consensus. Two participants dropped out before round 3. Subsequently, thirty participants were contacted to take part in round 3. In this final round, twenty-eight participants completed the survey (87.5%). A further six statements reached consensus (>70%). In total, 79 statements achieved consensus.

Main areas of agreement

Patient perspective and safety planning

Understanding how patients can contribute to their own safety emerged as a high research priority. Patient perspectives on medication safety, safety culture in those who self-harmed and the ways staff could help manage their self-harm were also considered a high priority (90.6%, 90.6% and 73.8% respectively). Experts had agreement on patient-centred research priorities including what constitutes good self-driven safety planning (90.6%), understanding patient’s perception of their own risk factors (71.4%) and an exploration into a personalised model of risk (81.3%). Consensus was also obtained on research exploring the patient perspective on staff violence on patients and research on ligature points (both 78.1%).

Physical health in mentally ill patients, mental health and physical health comparison and patient safety in the community

Experts indicated that exploring physical health adverse events in mental health patients should be a priority (81.3%). To a lesser extent, staff engagement with mental health patients about their physical health (75.0%) and comparison of physical and mental health hospitals on patient safety and errors (71.9% and 71.4% respectively) were seen as research priorities. Consensus was also achieved in a research priority exploring the

For peer review only - http://bmjopen.bmj.com/site/about/guidelines.xhtml

relationship between patients waiting to be seen in accident and emergency rooms and the link to general violence (78.1%).

Suicide prevention

Experts agreed that studies looking at suicide prevention interventions in large samples, marginal groups and outpatients were research priorities in the patient safety in mental health field (83.3%, 81.0% and 81.0% respectively). There was also a preference for research exploring suicide prevention in community samples. For example, the detection of people who are at risk of suicide but not in the mental health system; risk factors for suicide on discharge from hospital and a comparison between the community management, general hospital and psychiatric hospitals on their approach for suicide prevention were deemed future important research areas (78.6%, 78.1% and 71.4% respectively). A need to look at adverse events other than suicide in mentally ill patients was also evident (81.0%)

Safety culture and physical environment

There was clear preference for research on making psychiatric inpatient settings safer for patients. Priorities included the best approach to ensuring a safe environment in mental health units (85.7%), understanding and improving safety culture (83.3%) and identification of environmental factors that indicate a safe environment (81.0%). Likewise, experts wanted to know what environmental factors influence violent incidents and to observe adverse events on the wards (76.2% and 85.7% respectively). Explicit research priorities within this safety culture and environment section included an exploration of the relationship between therapeutic engagement and patient safety, general preventable errors and the safe alternatives to admitting someone to hospital, all of which gained a high-level consensus (81.0%, 78.2% and 81.0% respectively). The establishment of best practice guidelines for the design of inpatient settings was also strongly supported (87.5%).

Restraints

Experts agreed that research into the contributory factors to restraint, specifically in patients with physical health problems was a priority (85.7%). The prevalence and reasons for differences in restraint across units were also a priority (85.7%, 75.0% and 71.4% respectively). Two priorities focused on understanding the individual perspective in relation to restraint, including exploration of patient trauma after restraint (78.6%) and how people

For peer review only - http://bmjopen.bmj.com/site/about/guidelines.xhtml

feel about restraint in general (71.9%). Finally, experts agreed that research was needed to examine factors allowing for reduction in restrictive practice (90.5%) and alternatives to restraint (88.1%).

General safety, risk and violence management

Two research priorities that aimed to examine the nature and benefits of risk assessment (75.0% and 78.1% respectively) were supported by experts. Additionally, experts indicated positive and negative factors that influence violence management and more protective factors for violence in mental health patients were deemed necessary research areas (both 78.6%). Furthermore, experts agreed that the role of racism in relation to coercive interventions should be examined (71.4%). Other proposed priorities with strong agreement included staff decisions to admit patients presenting with self-harm behaviour (87.5%), what de-escalation should look like and how to evaluate it (87.5%) and exploration of staff attitudes on coercive practice (75.0%).

Psychological trauma

The expert group agreed that the mechanism of trauma and psychological harm associated with inpatient admission was a research priority (78.1%). There was also consensus that the patient experience of coercion in those who had experienced trauma should be examined (78.1%).

Children's safety

Experts indicated that research exploring the impact of adverse events for parents on the psychological wellbeing of their children was an important future research area. An understanding of predictive factors in self-harm and suicide in children and an exploration of a child's safety when parents with mental health problems become unwell both achieved consensus (83.3% and 88.1% respectively).

DISCUSSION

Main results and comparison to other studies

For peer review only - http://bmjopen.bmj.com/site/about/guidelines.xhtml

This is the first international Delphi study exploring research priorities in patient safety in mental health from both the academic and service user perspective. Sixty-eight per cent of statements were identified as future research priorities in patient safety in mental health (n=79). The top six research priority areas are shown in box 1; half of these areas were identified by patients for consideration. This suggests a need to shift the thinking about patient safety in mental health services to that of acknowledging the patient voice in relation to their own care and safety. This is in line with other clinical domains that have had success with patient involvement in achieving safer services. For example, some interventions that centre on error prevention through promotion of complex behavioural change and patient involvement have been deemed effective

Thirty-two percent of statements were not seen as priorities. Two research priority statements that concerned research identifying specific places of safety in the community had borderline consensus >65%; but in general, patient safety in community mental health settings were not seen to be a research priority. This is surprising given that most mental health care takes place in the community. Also surprising, considering that this was an international study with respondents from the USA, was the finding that participants disagreed/ strongly disagreed that access to guns in the community after discharge after discharge and omissions in prescriptions were research priorities (37.5% and 34.4%). This may be because the majority of survey experts were from countries where gun violence may not be prevalent in the general community, including among mental health patients.

Research on medication safety is seen as a priority in physical healthcare safety [17]. As such, we expected medication safety to also be identified as a research priority in mental health care; however, this was not the case. Although ‘patient perspectives on medication safety’ had strong consensus (Box 1), the remaining statements related to medication safety were not considered research priorities. Medication safety was not seen as a research priority overall; however, this may have been because the statements focused on minority groups including the elderly and patients with a dual-diagnosis and therefore the statements may not have been applicable to most experts, especially service users.

Box 1: Top six future research priorities that achieved the highest consensus

- Patient contributions to their own safety
- The patient perspective on medication safety
- Perspectives on safety culture in patients who self-harm

For peer review only - http://bmjopen.bmj.com/site/about/guidelines.xhtml

- Good self-driven individualised safety planning
- Safety plans and safety improvement
- Factors in allowing reduction in restrictive practice including restraint and seclusion

In general, statements that proposed longitudinal trials and research focusing on Safewards did not achieve consensus. It is possible that as longitudinal studies are deemed expensive and have added temporal demands [18], academic experts felt they would require a large programme grant and therefore were unattainable as a timely research priority. It is also possible experts felt de-escalation techniques may have had some focus in recent years [5,19] and therefore other areas required more focus going forward. Specifically, the identification of positive, protective and negative factors for violence were still deemed necessary research areas.

Some areas were not endorsed as priorities by the semi-structured interviews, including failure to establish diagnosis, the correct diagnosis and to deliver appropriate treatment. In addition, delays in accessing appropriate care because a bed is not available or service is unavailable, were also not mentioned. This is surprising because it has been considered an important barrier to improving mental healthcare service provision [20]; however, it is possible that experts considered this a patient care or quality improvement issue and not patient safety.

Strengths and limitations

This is the first Delphi study to collate research priorities across *all* aspects of patient safety in mental health internationally, over four continents. A survey sample which was notably larger than another Delphi study in the patient safety-mental health field [7] was obtained. A key strength is the involvement of service user experts in both the semi-structured interviews and survey stages. Another key strength is that all but one interviewed academic experts took part in the survey. The overall survey response rate was good and increased rather than decreased over each round (round 2, 76.2%; round 3, 87.5%).

However, there are some methodological limitations. Firstly, experts were mainly from Europe, the United States and other high income countries; therefore, the established set of

For peer review only - http://bmjopen.bmj.com/site/about/guidelines.xhtml

research priorities may only reflect a research agenda for developed countries. Secondly, only English speaking women service users participated in the interviews and therefore priorities may not be generalizable to the wider service user population. Thirdly, it was not easy to define an academic expert in the patient safety *and* mental health field; unlike another expert consensus building study [9]. This was because the patient safety in mental health field is still relatively new with experts likely to identify themselves as experts in mental health rather than patient safety. It is therefore possible academic experts were missed, unlike in another Delphi study [8]. Fourthly, experts who took part in the survey were not asked about their awareness of existing research in the area. Therefore, experts, particularly service users, could have recommended research that had already been covered. Furthermore, the survey did offer an opportunity to suggest additional areas for consideration that were not identified from the interviews. Finally, participant fatigue was a possible contributory factor towards latter statement completion, particularly due to the large amount of statements (n=117).

Implications

This Delphi study should inform the research agenda for patient safety in mental health field, specifically in the UK, US and other developed countries. It is recommended that academics wishing to pursue research in these areas conduct a comprehensive literature review pertaining to each priority area as a first step. Subsequent studies representing each priority area are then needed to target specific research areas, study types and methodologies. The research findings from these studies should be included in future policy and practice.

This study has included the service user 'voice' when identifying research priorities in patient safety in mental health field. Following this, it would be useful to co-create future research with service users, their families and carers, psychiatric nurses and other mental healthcare professionals with the aim of informing studies with an expert perspective. This is in line with current guidance and the increased expectation to involve a range of experts, including service user experts in the design, production and dissemination of research [21].

CONCLUSION

This is the first expert consensus study to identify research priorities in patient safety in the mental health field. Experts identified what they considered to be the most important areas for future research; these include the patient perspective on their mental healthcare,

For peer review only - http://bmjopen.bmj.com/site/about/guidelines.xhtml

including medication safety, safety planning and self-harm management. Whilst this Delphi study shows consensus in several areas, not all of these priorities are firmly on the research agenda for patient safety in mental health.

To establish patient safety in mental health as a research priority in its own right, prospective academic research programmes with appropriate funding are required. This will encourage research that helps detect deterioration of mental health, develops safety interventions and generally improves safety. Whilst mental healthcare provision is increasingly seen as a priority, there is still work to be done; the safety of mental health patients must have parity with patient safety within the physical health field.

Authorship/contribution statement

LD drafted and finalised the protocol, designed the study, led the data collection and analysis and interpreted the data. LD drafted the manuscript and is also the corresponding author.

KM advised on clinical definitions, mental health content interpretation and intellectual content. KM was involved in drafting the manuscript and approved the final version to be published.

BT contributed to data collection, was involved in drafting the manuscript and approved the final version to be published.

SR was involved in drafting the manuscript and approved the final version to be published.

SA advised on clinical definitions, critically reviewed intellectual content and was involved in the drafting and critical appraisal of the manuscript. SA also approved the final manuscript.

AD approved the final manuscript to be published.

SAr contributed to designing the protocol, advised on service user recruitment and critically reviewed intellectual content. SAr was also involved in drafting and critical appraisal of the manuscript.

The authors thank Dr Martine Nurek for her analytic support and all experts for their participation in this study.

Ethics approval

The study was approved by the Imperial College Research Ethics Committee.

Data sharing statement

No additional data are available.

For peer review only - http://bmjopen.bmj.com/site/about/guidelines.xhtml

For peer review only

Figures

Figure 1: Delphi diagram of study process

For peer review only

For peer review only - http://bmjopen.bmj.com/site/about/guidelines.xhtml

REFERENCES

1 Brickell TA, Nicholls TL, Procyshyn RM, *et al. Patient safety in mental health*. Edmonton: Canadian Patient Safety Institute and Ontario Hospital Association 2009. http://www.patientsafetyinstitute.ca/english/research/commissionedresearch/mentalhealthandpatientsafety/documents/mental health paper.pdf

2 Brickell T, McLean C. Emerging issues and challenges for improving patient safety in mental health: a qualitative analysis of expert perspectives. *J Patient Saf* 2011;**7**:39–44.

3 The Guardian. NHS mental health funding is still lagging behind, says report. 2016.https://www.theguardian.com/society/2016/may/09/nhs-mental-health-funding-is-still-lagging-behind-says-report (accessed 26 Apr 2017).

4 Prince M, Patel V, Saxena S, *et al.* No health without mental health. *Lancet* 2007;**370**:859–77.

5 Bowers L. Safewards: a new model of conflict and containment on psychiatric wards. *J Psychiatr Ment Health Nurs* 2014;**21**:499–508.

6 HM Government, Department of Health. Preventing suicide in England: A cross-government outcomes strategy to save lives. 2012. www.dh.gov.uk/ (accessed 13 Aug 2017).

7 Mascherek AC, Schwappach DLB. Patient safety priorities in mental healthcare in Switzerland: a modified Delphi study. *BMJ Open* 2016;**6**:e011494.

8 Cowman S, Björkdahl A, Clarke E, *et al.* A descriptive survey study of violence management and priorities among psychiatric staff in mental health services, across seventeen european countries. *BMC Health Serv Res* 2017;**17**.

9 Howell A-M, Burns EM, Hull L, *et al.* International recommendations for national patient safety incident reporting systems: an expert Delphi consensus-building process. *BMJ Qual Saf* 2016;bmjqs-2015-004456.

10 Linstone HA, Turoff M. The Delphi Method - Techniques and applications. *delphi method - Tech Appl* 2002;:1–616.

11 Kouyoumdjian FG, Schuler A, Mcisaac KE, *et al.* Using a Delphi process to define priorities for prison health research in Canada. *BMJ Open* 2016;**6**:1–8.

For peer review only - http://bmjopen.bmj.com/site/about/guidelines.xhtml

12 Owens C, Ley A, Aitken P. Do different stakeholder groups share mental health research priorities? A four-arm Delphi study. *Heal Expect* 2008;**11**:418–31.

13 Bissett M, Ot B. Occupational Therapy Research Priorities in Mental Health. *Occup Ther Heal Care* 2001;**14**:1–19.

14 D'Lima D, Archer S, Thibaut BI, *et al.* A systematic review of patient safety in mental health: a protocol based on the inpatient setting. *Syst Rev* 2016;**5**:203.

15 Fink A, Kosecoff J, Chassin M, *et al.* Consensus Methods: Characteristics and Guidelines for Use. http://ajph.aphapublications.org/doi/pdf/10.2105/AJPH.74.9.979 (accessed 21 Jul 2017).

16 Hasson F, Keeney S, McKenna H. Research guidelines for the Delphi survey technique. *J Adv Nurs* 2000;**32**:1008–15.

17 World Health Organisation (WHO). Global Priorities for Research in Patient Safety (first edition). 2008. http://www.who.int/patientsafety/research/priorities/global_priorities_patient_safety_research.pdf (accessed 21 Jul 2017).

18 Caruana EJ, Roman M, Hernández-Sánchez J, *et al.* Longitudinal studies. *J Thorac Dis* 2015;**7**:E537–40.

19 Bowers L, James K, Quirk A, *et al.* Reducing conflict and containment rates on acute psychiatric wards: The Safewards cluster randomised controlled trial. *Int J Nurs Stud* 2015;**52**:1412–22.

20 British Medical Association. Breaking down barriers – the challenge of improving mental health outcomes. London: 2017.

21 Omeni E, Barnes M, MacDonald D, *et al.* Service user involvement: impact and participation: a survey of service user and staff perspectives. *BMC Health Serv Res* 2014;**14**:491.

For peer review only - http://bmjopen.bmj.com/site/about/guidelines.xhtml

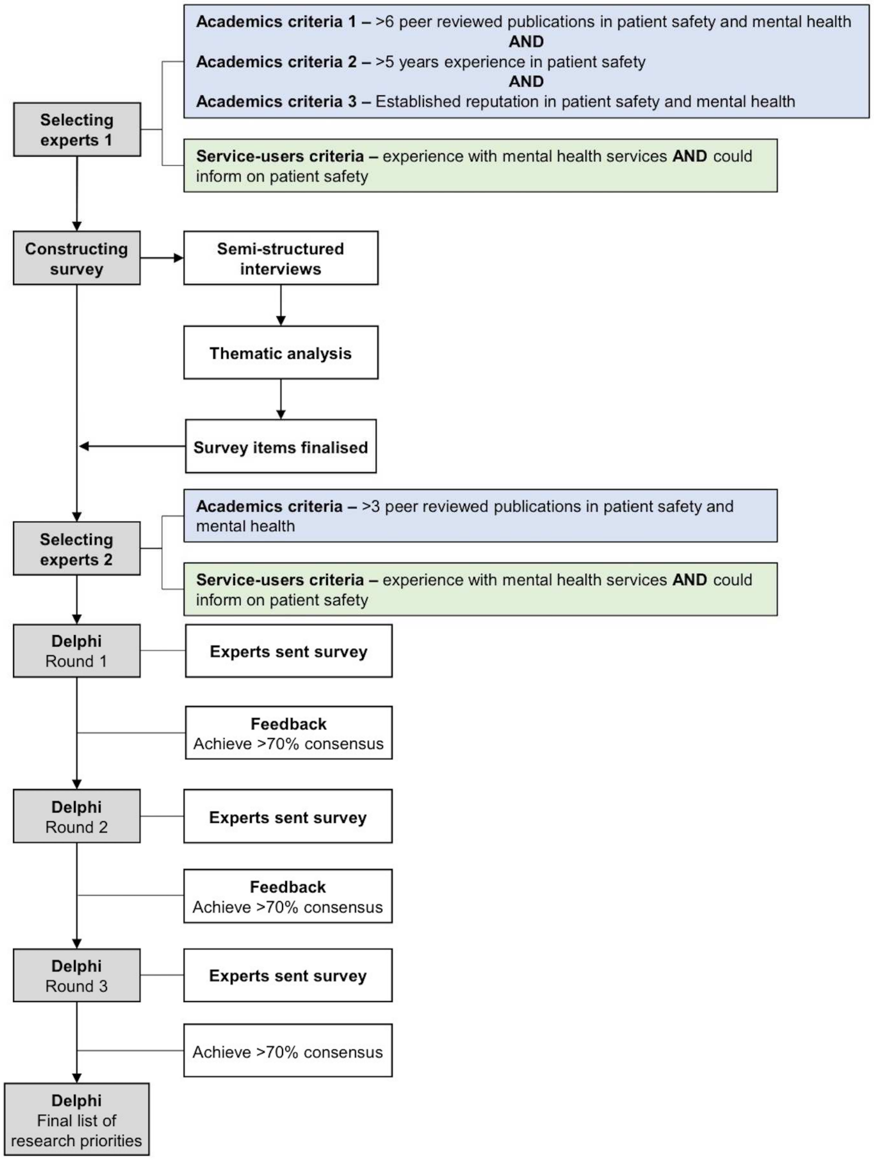

Figure 1: Delphi diagram of study process

396x529mm (300 x 300 DPI)

Supplementary file 1

Table 1: Research priorities for patient safety in mental health

No.	Research priority	% Consensus (n=79)
**Governance and improvement**		
**1**	To gain consensus on guidelines on how patient observation should occur and keep patients safe	78.6%
**Restraint**		
**6**	To use quality research to explore how people feel about restraint	71.9%
**7**	To identify important factors in allowing reduction in restrictive practices including restraint and seclusion	90.5%
**8**	To identify the main contributory factors in the use of restraint	85.7%
**9**	To establish the prevalence of restraint and reasons for differences among units	71.4%
**11**	To explore patients' trauma after restraint	78.6%
**12**	To understand why people die from restraint	71.4%
**13**	To identify alternatives to restraint	88.1%
**Children and patient safety**		
**15**	To understand how problems arise and escalate related to self-harm and suicide in children	83.3%
**16**	To explore children's safety when parents with mental health problems become unwell	88.1%
**17**	To explore preventing other harm and secondary damage to children	81.0%
**General safety**		
**18**	To conduct an observational study of adverse events on psychiatric units	85.7%
**19**	To identify what errors or adverse events happen as patients' interface between the medical and mental health system	76.2%
**20**	To investigate preventable errors or adverse events in general in mental health patients	78.2%
**21**	To explore the relationship between therapeutic engagement and patient safety	81.0%

For peer review only

**23**	To explore why people disengage with services on discharge from a mental health unit	73.8%
**24**	To identify the important factors on discharge to keep people safe and comparison across services	88.1%
**25**	To investigate the safe alternatives to admitting someone to hospital	81.0%
**26**	To examine dual diagnosis patients falling through the gap and what can be done to prevent this	71.4%
**Suicide prevention**		
**27**	To conduct a well-controlled study interventions to reduce suicide in outpatients	81.0%
**28**	To compare and contrast community approach, hospital approach and mental health approach in their attempt to reduce suicide	71.4%
**29**	To establish the effectiveness of suicide prevention interventions in large patient samples	83.3%
**30**	To examine suicide prevention in marginal groups	81.0%
**31**	To identify risk factors for suicide on discharge from hospital	78.6%
**32**	To detect people who are at risk of suicide but are not in the mental health system	78.1%
**33**	To identify adverse events that happen to mental health patients above and beyond suicide	81.0%
**Safe environments and culture**		
**39**	To examine the influence of the environment on violent behaviour	76.2%
**40**	To identify environmental factors that indicate a safe environment	81.0%
**41**	To establish the best approach to ensuring safe environments in mental health units	85.7%
**42**	To examine the management of a safe and therapeutic inpatient space	81.0%
**43**	To understand the cultural aspects of safety within an organisation or ward and the major influences in improving the safety culture	83.3%
**44**	To explore the climate and cultural tool use in relation to what influences patient safety culture	75.0%
**45**	To establish best practice guideline for design of an inpatient setting	87.5%
**46**	To identify what constitutes a safer acute ward build	73.8%
**Violence management**		
**47**	To identify positive and negative factors that influence violence management	78.6%

For peer review only

**48**	To understand what prevents violence in patients with mental health conditions	78.6%
**Risk assessment**		
**49**	To understand the nature of the concept of risk assessment	75.0%
**50**	To examine the benefits of a good risk assessments system versus someone who had no risk assessment	78.1%
**Patient perspective or patient centred**		
**53**	To explore the patient perspectives on reasons for their self-harm behaviour	73.8%
**54**	To explore patient perspectives on ways staff can help them manage their self-harm behaviour	73.8%
**56**	To explore the patient perspective on medication safety	90.6%
**57**	To explore patient perspectives on ligature points in an inpatient environment	78.1%
**58**	To explore patient perspectives on their own safety	73.8%
**59**	To explore staff and patient perspective on what determines a safer culture in inpatient setting	87.5%
**60**	To explore perspectives on safety culture in patients who self-harm	90.6%
**61**	To explore patient experience of staff assault on patients	78.1%
**62**	To explore patient experience of complaints after coercive intervention	75.0%
**Decision making and safety**		
**63**	To investigate staff decisions to admit patients presenting with self-harm behaviour	81.3%
**Demographic studies and safety**		
**65**	To evaluate same sex wards compared to mixed sex wards in relation to patient safety	78.1%
**66**	To examine the role of racism in the mental health system in relation to coercive interventions	71.4%
**Psychological trauma**		
**67**	To examine mechanism of trauma and psychological harm associated with inpatient admission	78.1%
**68**	To explore patient experience of coercion in those who've experienced trauma	78.1%
**Safety intervention studies**		

For peer review only

**73**	To conduct trial of different interventions to reduce patient safety incidents compared with other interventions	71.4%
**75**	To establish de-escalation should constitute and how to evaluate it	87.5%
**Safety plans and self-management**		
**76**	To understand specific things patients know regarding their own risk	71.4%
**77**	To identify what constitutes good self-driven individualised safety planning	90.6%
**78**	To conduct a large trial on whether safety plans could improve safety	90.6%
**79**	To investigate personalised model of risk	81.3%
**80**	To understand how patients themselves can contribute to their own safety	100.0%
**81**	To examine the self-management of service users of their own recovery	84.4%
**Staff perspective**		
**89**	To explore the attitudes of staff and its impact on coercive practice	75.0%
**90**	To identify future training of staff in coercive practice	71.9%
**Patients in the community and their safety**		
**95**	To explore what constitutes a place of safety in the community for mentally ill patients	75.0%
**98**	To examine mental health safety in the community	81.3%
**101**	To explore waiting to be seen in accident and emergency rooms and the link to violence	78.1%
**102**	To understand what support is available after discharge	71.9%
**Physical health in mentally ill patients**		
**103**	To examine how improving the quality of life and the physical health of people with severe mental illness	71.4%
**104**	To examine mental health staff engagement with inpatients on their physical health	75.0%
**105**	To compare and contrast physical health hospitals and mental health hospital take on patient safety	71.9%
**107**	To explore of physical health adverse events in mental health patients	81.3%
**108**	Factors related to restraint in mental health patients with physical health problems	71.9%
**109**	Relationship between lack of physical activity in acute settings and dangerous behaviour	75.0%
**Safety in general hospitals for mentally ill patients**		

For peer review only

**111**	To examine safety in mental health patients diagnosed with mental health condition in general medical hospitals	71.9%
**112**	To compare and contrast errors and adverse events in general medical hospitals in patients with mental illness and those without mental illness	71.4%
**Death**		
**115**	To identify reasons for early death in patients with mental health problems	78.1%

**Priorities in bold italics did not come to a consensus*

For peer review only

Supplementary file 2

Table 2: Research priorities for patient safety in mental health that did not achieve consensus

No.	Research priority	% Consensus (n=38)
**Governance and improvement**		
**2**	*To examine clinical and cost effectiveness of early patient discharges*	***64.3%***
**3**	*To conduct longitudinal studies on patient records to monitor and improve patient safety*	***57.1%***
**Models of safety**		
**4**	*To understand traditional models of safety and how mental health care works within the model*	***69.0%***
**5**	*To explore philosophical perception of patient safety on patient's rights*	***46.9%***
**Restraint**		
**10**	*To employ a naturalistic study to compare and contrast use of restraint intervention in different countries*	***65.6%***
**14**	*To explore the use of restraint in Black-British men*	***53.6%***
**General safety**		
**22**	*To explore whether mental health first aid has saved a life*	***60.7%***
**Dual diagnosis and safety**		
**34**	*To explore dual diagnosis of substance misuse among psychiatric patients*	***59.4%***
**35**	*To examine the impact on the health and behaviour in dual diagnosed patients*	***53.1%***
**36**	*To conduct a scoping review of range of services available for dual diagnosis patients*	***53.1%***
**37**	*To explore the relationship between specific mental health conditions and substance misuse*	***62.5%***
**38**	*To explore safety incidents in dual diagnosed patients*	***65.6%***
**Research methodology only**		
**51**	*To use mixed methodologies as an research methodology to use*	***57.1%***

For peer review only - http://bmjopen.bmj.com/site/about/guidelines.xhtml
For peer review only

**52**	*To use qualitative research methods to explore priorities*	***59.4%***
**Patient perspective or patient centred**		
**55**	*To explore subjective patient experience of coercion*	***67.9%***
**Demographic studies and safety**		
**64**	*To explore LGBT communities with mental health problems and safety incidents*	***59.4%***
**Psychological trauma**		
**69**	*To observe subjective distress and satisfaction in the short-term*	***40.6%***
**Safety intervention studies**		
**70**	*To conduct large longitudinal service wide intervention trial*	***60.7%***
**71**	*To conduct a stepped wedge randomised controlled trial of Safewards*	***64.3%***
**72**	*To implement six core strategies in different settings across countries to understanding outcomes*	***68.8%***
**74**	*To conduct big intervention study on how a patient autonomy model of patient care can be safer*	***60.7%***
**Medication safety**		
**82**	*To examine the impact of medication on the patient*	***59.4%***
**83**	*To conduct randomised controlled observational studies of tapering and withdrawing medication*	***53.1%***
**84**	*To understand about the interaction of multiple medications in relation to safety*	***53.1%***
**85**	*To examine prescription medication assessments in elderly patients with mental health issues*	***50.0%***
**86**	*To explore the polypharmacy and medication management of dual diagnosed patients*	***50.0%***
**87**	*To examine the safety of medication by injection*	***46.9%***
**88**	*To examine the omissions in prescriptions*	***34.4%***
**Staff perspective**		
**91**	*To understand why staff react to events on ward in certain ways*	***65.5%***
**92**	*To explore the physician perception of mental health patients and how they should be managed*	***64.3%***
**93**	*To explore staff attitudes on levels of violent behaviour*	***68.8%***
**Patients in the community and their safety**		
**94**	*To identify places of safety for mentally ill patients in the community*	***68.8%***
**96**	*To examine the impact of discharged patients back into the community with access to guns*	***37.5%***

For peer review only

**97**	*To understand how the system takes care of the mental health patient in the community*	***64.3%***
**99**	*To explore safety concerns in the community when patients are withdrawn*	***57.1%***
**100**	*To assess whether crisis cafes the answer to saving lives rather than being detained in hospital*	***65.6%***
**Physical health in mentally ill patients**		
**106**	*To examine food as an addiction in mentally ill patients*	***40.6%***
**110**	*Treatment from staff in mental health patients in emergency or medical situations*	***65.6%***
**Miscellaneous**		
**113**	*To explore patient personal space and acceptability of body examination*	***43.8%***
**114**	*To conduct a large European study looking at best practice across numerous countries*	***59.4%***
**Service user involvement**		
**116**	*To use senior service user researcher to lead mental health and safety work*	***56.3%***
**Incident reporting**		
**117**	*To compare and contrast recorded and actual observed patient safety incidents in inpatient settings*	***68.8%***

**Priorities in bold italics did not come to a consensus*

For peer review only
